# Lacrimal gland Alterations and the Effect of artesunate on experimental induced diabetes rat models and related mechanisms

**DOI:** 10.1038/s41598-024-61550-0

**Published:** 2024-05-31

**Authors:** Girju Rajbanshi, Wei Li, Xiaolin Nong, Yi Li, Dongxiao Nong

**Affiliations:** 1 Department of Oral & Maxillofacial Surgery, College of Stomatology, Hospital of Stomatology, Guangxi Medical University, 10 Shuangyong Road, Nanning, 530021 Guangxi China; 2 Department of Pediatrics Dentistry & Preventive Dentistry, College of Stomatology, Guangxi Medical University, Nanning, 530021 Guangxi China; 3 Guangxi Key Laboratory of Oral and Maxillofacial Rehabilitation and Reconstruction, Nanning, 530021 Guangxi China; 4 Department of Oral & Maxillofacial Surgery, College of Stomatology, Hospital of Stomatology, Guangxi Medical University, 10 Shuangyong Road Nanning, Guangxi, 530021 China; 5 Department of Otorhinolaryngology-Head and Neck Surgery, First Affiliated Hospital of Guangxi Medical University, Guangxi Medical University, Nanning, 530021 Guangxi China

**Keywords:** Type 1 diabetes mellitus, Lacrimal gland, Eyeball, Dry eye syndrome, Artesunate, Drug discovery, Endocrinology

## Abstract

Diabetic patients are at high risk of developing lacrimal gland dysfunction, and the antimalarial drug artesunate (ART) was recently used to induce experimental-induced diabetes mellitus. This study’s objective is to investigate the lacrimal gland alteration and the effect of ART on experimentally induced diabetes rat models and its related mechanisms. Forty rats were divided into five groups (8 rats/group): healthy control group (HC), diabetic group (DM), 50 mg/kg ART intervention diabetic group [DM + ART (50 mg/kg)], 100 mg/kg ART intervention diabetic group [DM + ART (100 mg/kg)] and 6 U/kg Insulin intervention diabetic group (DM + INS). The morphology of the eyeball and lacrimal gland tissues was determined using hematoxylin and eosin staining. In addition, external lacrimal glands were harvested for electronic microscopic examination, NFκB1, and TNF-α protein expression evaluation by immunohistochemistry and mRNA expression analysis by RT-PCR. Histopathological and ultrastructural changes suggest ART intervention has an improved structural effect. Protein expression of NFκB1 in the DM + ART (100 mg/kg) group was decreased. TNF-α significantly decreased in the DM + ART (50 mg/kg) and insulin groups. We concluded that ART improves structural changes in a lacrimal gland in diabetic rats. The present study provides further evidence of the therapeutic effect of ART on the lacrimal gland of diabetic rats by decreasing the expression of NFκB1 and TNF-α.

## Introduction

In recent years, there has been a significant increase in the global prevalence of diabetes mellitus, leading to a higher number of individuals experiencing different complications related to diabetes, such as ocular surface diseases^1^. These conditions can have a detrimental impact on the individual’s overall well-being. Diabetes mellitus has the potential to cause a range of eye-related problems, including diabetic retinopathy, glaucoma, cataracts, and conditions on the surface of the eye^2^. Ophthalmologists are increasingly recognizing the impact of diabetic ocular surface complications, prompting a growing focus on understanding the underlying mechanisms and identifying potential approaches for treating these complications^3^.

The major risks associated with diabetic patients include conditions such as superficial punctuate keratitis, recurrent corneal erosions, persistent epithelial defects, and microbial keratitis. These signs and symptoms are believed to be linked to dysfunction of the lacrimal gland, leading to a condition known as dry eye characterized by an unstable tear film and ocular surface epithelial disease, inflammation, lacrimal gland inflammation, and secretory dysfunction. Studies have proven that nearly 50% of diabetic patients experience tear-deficient dry eye disease (TDDE) or aqueous dry eye disease (DED)^4^. Understanding and addressing these risks is essential to provide comprehensive care for diabetic individuals. If left untreated severe DED may cause pain, corneal ulceration, and potential loss of vision^5,6^. In diabetes mellitus (DM), chronic hyperglycemia, oxidative stress, nerve alterations, and disturbance in insulin function may be fundamental aspects in the progression of changes in the ocular surface (OS) and impairment of the lacrimal gland (LG). However, the exact mechanism behind these changes is not thoroughly understood^7^.

537 million people suffer from diabetes worldwide and dry eye is a significant complication of diabetes up to 54.3%. The estimated prevalence of diabetes in China increased from 10.9% in 2013 to 12.4% in 2018 and the estimated prevalence of prediabetes in the same country was 35.7% in 2013 and 38.1% in 2018 respectively^8^. Research on the epidemiology of diabetic ocular surface complications under DM conditions has focused on dry eye disease (DED), with 1360 patients in the Beixinjing community exhibiting a 17.5% incidence of diabetic dry eye disorder^9^. Tear film instability leads to dry eye, caused by an imbalance in tear secretion or evaporation. This condition can result in discomfort and potential damage to the surface and structure of the eye if untreated^10^. Therefore, it is important to examine the ocular surface regularly to discover dry eyes early and avoid visual complications.

Artesunate (ART) is a vital derivative of artemisinin extracted from the natural herb Artemisia annua. It has a pharmacological profile with water solubility and high oral bioavailability. According to the World Health Organization's List of Essential Medicines, it is viewed as a secure and high-quality anti-malarial drug^11^. Furthermore, ART possesses anti-viral, anti-inflammatory activity, antioxidative, anti-cancer impact, and anti-glucose effect^12^. ART induced the conversion of α cells to functional β-like cells in vitro and accelerated pancreatic islet size in mice. Furthermore, the investigation validated the feasibility of ART as an ability to defend beta cells towards IL-1β-induced impairment of Glucose-stimulates insulin secretion (GSIS), and the feasible molecular mechanism had been conducted^13^. Our previous studies also confirmed that artesunate could significantly decrease blood glucose and improve cardiovascular complication development^14^. Li et al. have shown that ART can inhibit NF-κB-mediated inflammation signaling pathways and reduce blood glucose activity^15^.

Much of our current understanding of the complications that diabetes can cause in the eyes has mainly focused on the lacrimal gland. However, the ocular surface is another crucial structure that is damaged by diabetes. Diabetic ocular surface complications can cause more serious side effects than dry eye syndrome. Therefore, in this study, we aimed to investigate the role of Nuclear factor kappa B (NF-κB) and tumor necrosis factor-alpha (TNF-α) signaling pathway in a type 1 diabetes rat model. We explored the potential effects of ART, known for its anti-inflammatory, antifibrotic, and blood glucose-lowering properties of ART on the NF-κB/ TNF-α signaling pathway to improve dry eye in rats with diabetes. Understanding this could provide valuable insights into the treatment of dry eye syndrome in patients with diabetes mellitus.

## Results

We developed an animal model of experimentally induced diabetes by intraperitoneal injection of STZ (60 mg/kg/ body weight). In the present study, animals developed eye problems as they developed glucose intolerance and diabetes. The glycemic level used to establish the diagnosis of diabetes was ≥ 16.7 mmol/L^16^.

## Blood glucose and body weight monitoring results of rats in each group

**Figure 1:**
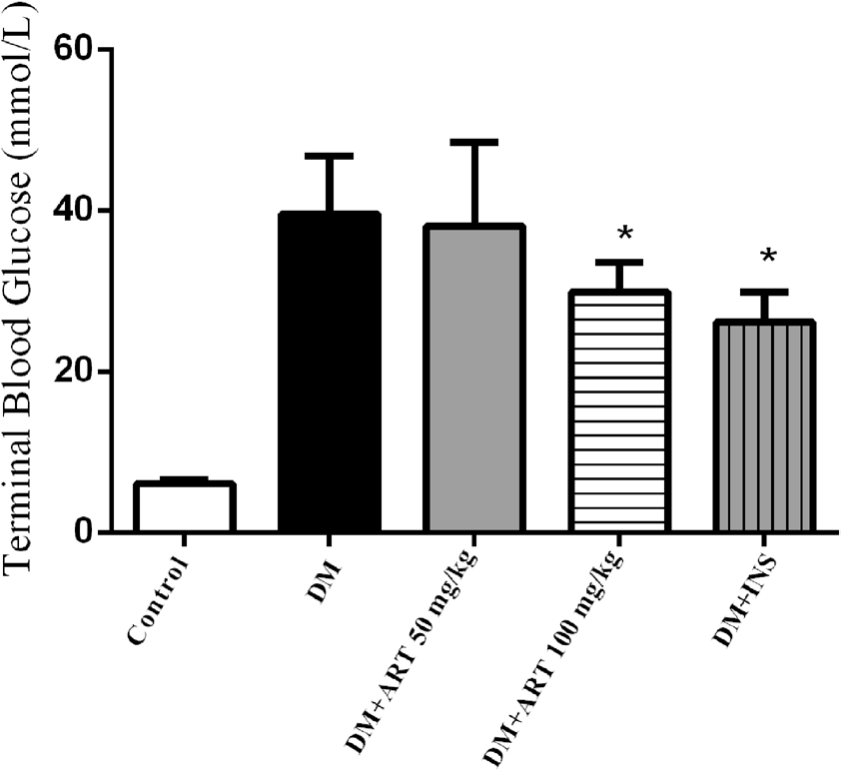
Terminal Blood glucose level of each group, Data are presented as mean ± SD, *P < 0.05 compared with the healthy control group.

**Figure 2:**
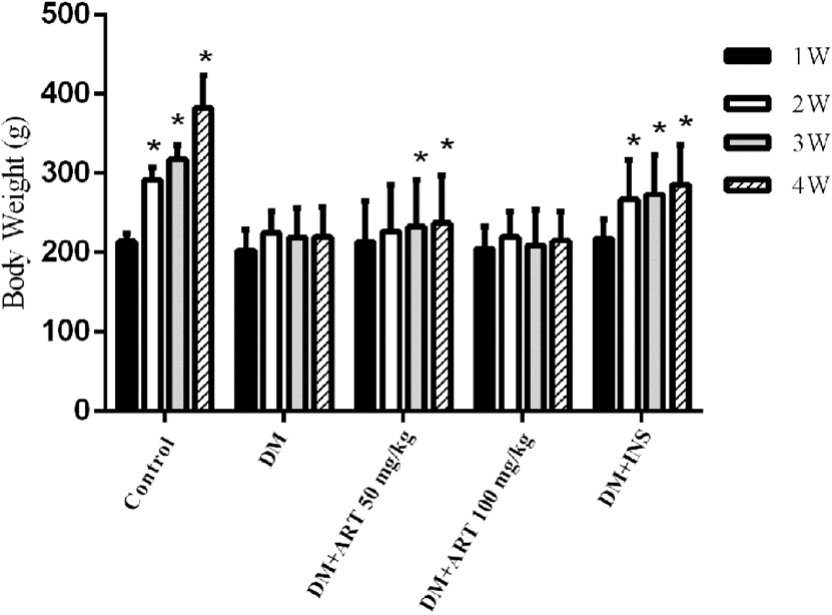
Result of body weight of each group, the body weight of each group at different time points was compared with that of DM group. Data are presented as mean ± SD, *P < 0.05 compared with the healthy control group.

(Figure 1), After the start of the experiment, the average blood sugar levels of the diabetes and intervention groups were significantly higher than those of the normal group and stably higher than 16.7 mmol/L. There was no statistically significant difference in fasting blood glucose levels between each intervention group and the DM group. As shown in Table 1 (Fig. 2) in the first week after the start of the experiment, there was no significant difference in body weight between the groups. With the prolongation of time, the body weight of the healthy control group increased rapidly and was significantly higher than that of other groups (^*^P < 0.05). Compared with the healthy control group, the weight growth rate of the diabetic group was significantly decreased. Among the intervention groups, the insulin group had the fastest weight gain, and DM + ART (50 mg/kg) also had a considerable increase in body weight at the end of the experiment. Both groups were significantly higher than the diabetes non-intervention group (^*^P < 0.05).

**Table 1:** Dynamic monitoring results of body weight of rats in each group (n = 8).

Group	$\overline{x}$± s			
	1W	2W	3W	4W
C	214.0 ± 10.0	291.4 ± 15.9*	317.5 ± 17.3*	382.6 ± 41.0*
DM	202.5 ± 26.7	224.6 ± 27.5	219.5 ± 36.8	219.7 ± 37.8
DM + ART 50	213.7 ± 51.2	226.9 ± 59.0	232.5 ± 59.2*	237.1 ± 60.8*
DM + ART 100	205.1 ± 27.7	219.9 ± 31.7	209.2 ± 45.3	214.6 ± 37.3
DM + INS	217.6 ± 24.4	267.3 ± 49.6*	273.4 ± 50.2*	285.6 ± 50.5*

*P < 0.05. Compared with the DM group at different time points in each group.

## Tear secretion, Weight of lacrimal gland, and lacrimal gland weight per body weight increased in the ART group compared to the type 1 diabetes mellitus group

**Figure 3:**
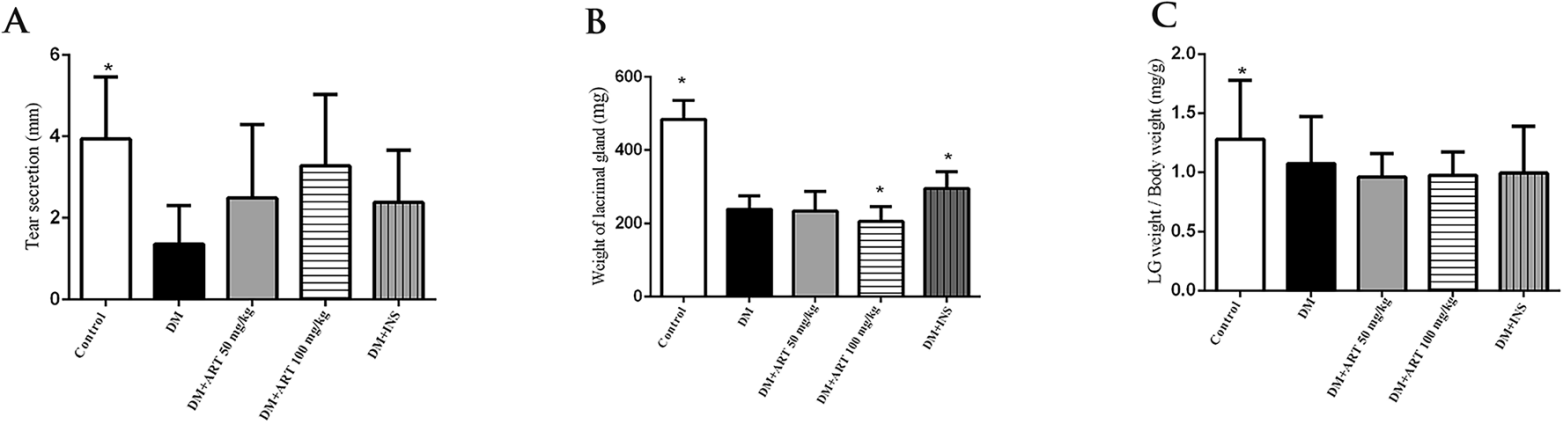
(**A**), (**B**), (**C**): At the end of the experiment, the result of tear secretion, the weight of lacrimal gland, and the weight of lacrimal gland/body weight ratio respectively of each group, Data are presented as mean ± SD, *P < 0.05 compared with the control group.

The phenol thread test was performed to assess tear production four weeks after the successful establishment of diabetic models. The tear secretion flow by phenol red thread test showed significantly reduced tear production in the diabetic group [1.362 ± 0.94 mm] compared with the healthy control group [3.937 ± 1.522 mm], (^*^P < 0.05). The tear secretion in DM + ART (100 mg/kg) intervention group [3.277 ± 1.750 mm] was increased compared to DM + ART ( 50 mg/kg )intervention group [2.488 ± 1.801] and DM + INS intervention group [2.377 ± 1.281]; (^*^P > 0.05). The average weight of the lacrimal gland in normal rats was (484.227 ± 51.86 mg) which was significantly higher than that in the diabetic group (238.085 ± 37.008 mg) (*P < 0.05). The weight of the lacrimal gland was significantly higher in DM + INS [295.177 ± 45.401] compared to DM + ART (50 mg/kg) [233.43 ± 53.75 mg] and DM + ART (100 mg/kg) [206.488 ± 38.147 mg/kg] (*P < 0.05). The lacrimal gland weight/body weight in the control group [1.23 ± 0.5 mg/g] was significantly higher than that in the diabetic group [1.07 ± 0.4 mg/g]. All intervention groups were significantly lower than the diabetes group (*P < 0.05) (Table 2) (Fig. 3A, B, C).

**Table 2:** Results on tear secretion flow, body weight, weight of lacrimal gland, and lacrimal gland weight/body weight in each group.

Parameters	$\overline{x}$± s				
	C	DM	DM + ART 50	DM + ART 100	DM + INS
Tear secretion (mm)	3.93 ± 1.52*	1.3 ± 0.9	2.4 ± 1.8	3.2 ± 1.7	2.3 ± 1.2
Body weight(g)	447.6 ± 41.0*	222.7 ± 31.8	242.1 ± 60.8*	211.6 ± 37.3	297.6 ± 50.5*
LG weight(mg)	484.2 ± 51.8	238 ± 37.0*	233.4 ± 53.7	206.4 ± 38.1*	295.1 ± 45.4*
LG weight/Body weight(mg/g)	1.28 ± 0.5*	1.07 ± 0.4	0.96 ± 0.2*	0.97 ± 0.2*	0.99 ± 0.4*

## Type 1 diabetes mellitus with ART and insulin treatment showed no significant changes in the eyes

**Figure 4:**
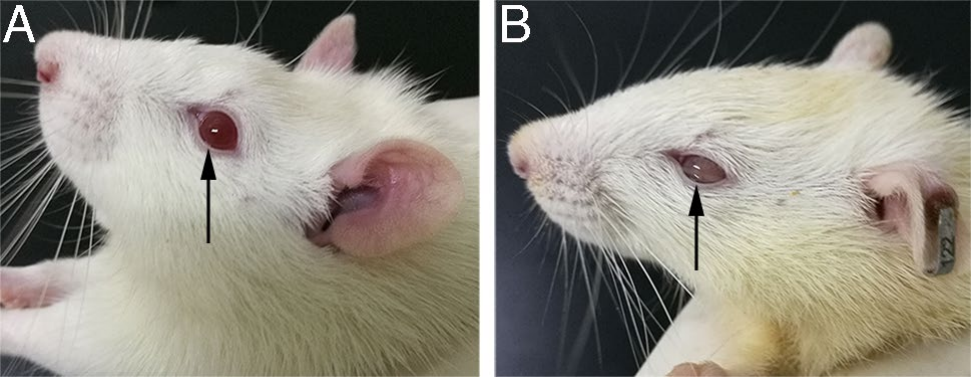
Diabetes-associated dry eye syndrome. Two experimental rats (**A**: control; **B**: diabetic rat).

Experimental animals developed dry eyes a few weeks after STZ administration as they developed glucose intolerance and diabetes. From slight corneal erosion to severe keratitis, there are apparent clinical signs of eye disease. During the experiment, the observation found that the eyeball of the control group was normal; light reflection was sensitive; and the surface of the cornea was moist (Fig. 4A). The eyes of DM rats and all other intervention groups were gray; the visual response was slow; the corneal surface was dry, and squinting was preferred (Fig. 4B). At the end of the experiment, suspected cataract changes and concomitant visual impairment were observed in the DM group’s eyes. In insulin and ART intervention groups, there were no significant changes.

## Corneal histopathology

**Figure 5:**
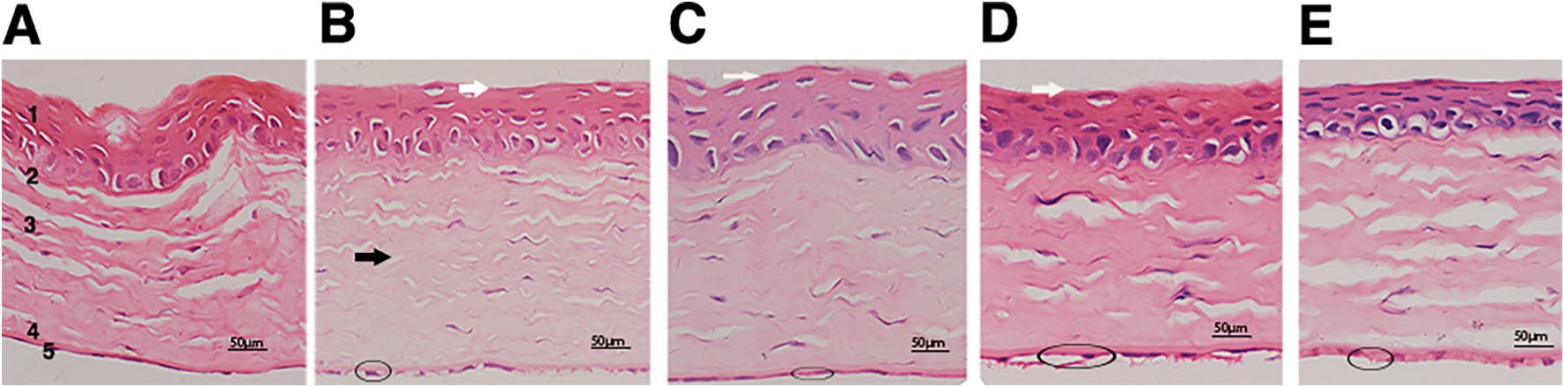
The structure of cornea histology by H&E staining. (**A**) H&E staining of the cornea from normal controls; (**B**) Diabetic (DM) group; (**C**) Artesunate (ART) 50 mg/kg intervention diabetic group; (**D**) Artesunate (ART) 100 mg/kg intervention diabetic group and (**E**) Insulin (INS) intervention diabetic group. 1. Corneal epithelium, 2. Bowman layer, 3. Corneal stroma, 4. Descemet’s membrane, 5. Monolayer corneal endothelial layer. The corneal stromal layers in the STZ-induced diabetic rats were decreased compared with those in the respective normal rats. ART 100 mg/kg and the Insulin intervention group have increased corneal stroma compared to the diabetic group. Note that the cornea of diabetic rats has epithelial defects and fragility, and abnormal collagen fibrils on the stroma compared to the control. DM + INS (**E**) and ART 100 mg/kg (**D**) intervention group had increased corneal stroma compared to the diabetic group. Scale bar = 50 µm. Original magnification 600 × (**A**–**E**).

In normal rat corneal tissue, the cornea has five layers: 1. corneal epithelium, 2. Bowman layer 3. Corneal stroma, 4. Descemet’s membrane, 5. Monolayer corneal endothelial layer. Hematoxylin and eosin (H&E) staining showed that compared with normal rats, increased corneal stromal cell infiltration and adhesion defects of corneal basal cells and Bowman’s membrane related to DM group. Abnormal collagen fibril bundles could be seen on stromal and increased pleomorphism and variability of cell area in the corneal endothelial layer of the diabetic group. The DM + INS and DM + ART (100 mg/kg) intervention group had increased corneal stromal compared to the diabetic group (Fig. 5).

## Lens histology: ART intervention increased the number of cell nuclei and decreased the number of vacuoles in the cortical region

**Figure 6:**
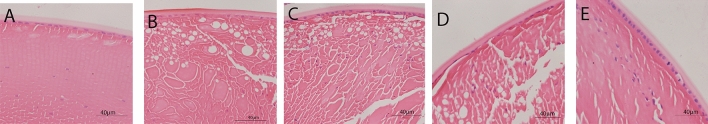
Histology of lens. In (**A**) healthy control group, (**B**) diabetic group, (**C**) DM + ART (50 mg/kg), (**D**) DM + ART ( 100 mg/kg ), (**E**) DM + INS (6 U/kg). In the control group, the following number indicates 1. Lens capsules and sub-capsular epithelium 2. Cortex of the lens. The number of cell nuclei of the lens on the epithelium layer (rectangle); and a large number of distinctive vacuolar changes in the cortical region (black arrow). Note that the lens of diabetic rats remarkably decreased cell nuclei and large vacuolar changes. DM + ART (100 mg/kg) (**D**) have increased cell nuclei and fewer vacuoles were observed in the cortical region. Scale bar = 40 µm. Original magnification 200 × (**A**–**E**).

The number of cell nuclei in the lens was remarkably decreased, and a large number of distinctive vacuolar changes in the cortical region was observed in the diabetic group compared to the control and ART intervention groups. In the DM + ART (100 mg/kg), the number of cell nuclei was increased significantly, and fewer vacuoles were observed in the cortical region (Fig. 6) compared to diabetic groups.

## Retina histology: ART intervention decreased the loss of the outer plexiform layer (OPL) compared to the diabetes mellitus group

**Figure 7:**
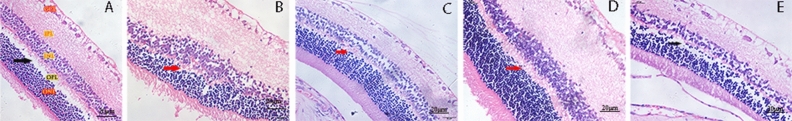
Histology of retina. In (**A**) healthy control group, (**B**) diabetic group, (**C**) DM + ART (50 mg/kg), (**D**) DM + ART (100 mg/kg), (**E**) DM + INS (6 U/kg). Different layers of retina GCL: ganglion cell layer; IPL: inner plexiform layer; INL: inner nuclear layer; OPL: outer plexiform layer; ONL: outer nuclear layer. Outer plexiform layer (OPL) (black arrow); loss of outer plexiform layer (OPL) (red arrow). Note that the lacrimal glands of diabetic rats have a loss of Outer plexiform layer (OPL) as compared to the control. DM + INS (**E**) has the formation of OPL. Scale bar = 20 µm.

The morphological changes were observed in the inner nuclear layer (INL), and outer nuclear layer (ONL), and degenerated ganglionic cells and partial loss of the outer plexiform layer (OPL) were observed in the diabetic group. In the DM + ART (50 mg/kg) and DM + ART (100 mg/kg) intervention groups, there was more loss of the outer plexiform layer (OPL) whereas there was more formation of the outer plexiform layer (OPL) in DM + INS intervention group (Fig. 7).

## Lacrimal gland histology: ART shows a normal acinar and lobular pattern with lymphocyte infiltration

**Figure 8:**
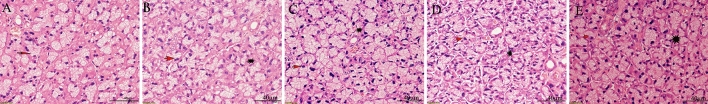
Histology of lacrimal gland. In (**A**) healthy control group, (**B**) diabetic group, (**C**) DM + ART (50 mg/kg), (**D**) DM + ART (100 mg/kg), (**E**) DM + INS (6 U/kg). Shape and size of acinar cells (red arrow); the infiltration of polymorphonuclear cells and lymphocyte cells (black star); Note that the lacrimal glands of diabetic rats irregularly, atrophied, and clustered acinar cells. Compared to control. DM + ART (50 mg/kg) (**C**) and DM + INS (**E**) intervention groups have unchanged lobular patterns and no acinar atrophy and fibrosis, inflammatory infiltration is more. Scale bar = 40 µm. Original magnification 400 × (A-E).

In the healthy control group, the normal lobular pattern was observed. There was no acinar atrophy or acinar fibrosis, and no significant lymphocyte infiltration was noted. The diabetic group revealed abnormal lobular patterns and disorganized acinar and ductal cell size and shape. The infiltration of polymorphonuclear cells and lymphocytes was present. In DM + ART (50 mg/kg) and DM + INS intervention groups the lobular pattern was normal and there were no acinar atrophy and fibrosis. The size and shape of the acinar and ductal cells were similar to the healthy control group, and were lymphocytes infiltrated. In the DM + ART (100 mg/kg) intervention group, a large number of lymphocytes were infiltrated in the lacrimal gland (Fig. 8).

## ART increased rER, mitochondrial cristae and but did not affect nucleus morphology

**Figure 9:**
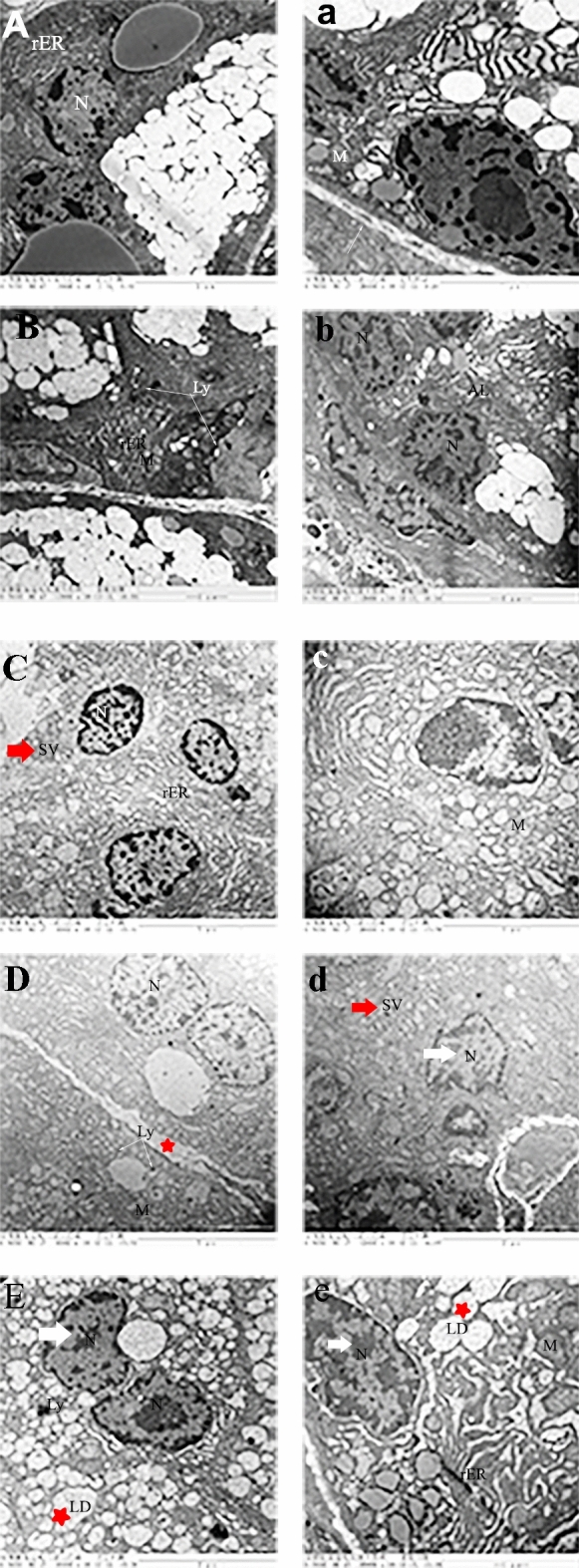
Ultrastructural changes of lacrimal gland cells in rats. In (**A**) healthy control group, (**B**) diabetic group, (**C**) DM + ART (50 mg/kg ), (**D**) DM + ART (100 mg/kg ), (E) DM + INS(6 U/kg). Nucleus (N), rough endoplasmic reticulum (rER), Mitochondria (M), Lysosome (Ly), Secretary vesicles (SV), lipid droplets (LD). Note that the lacrimal glands of diabetic rats have smaller secretory granules (red arrow), fragmentation and shrinkage of nuclei (white arrow); large number of lysosomes and lipid droplets in the basal cytoplasm (red star) compared to control.

To examine the secretory vesicle (SV) accumulation, mitochondrial, and nuclei alteration in detail, we analyzed the ultrastructural morphology of the lacrimal gland. In healthy control groups, the nucleus was round, with no phenotypic alterations of mitochondria, and there was abundant rough endoplasmic reticulum (rER). The secretory granules of the lacrimal glands of healthy rats were significantly larger, numerous, dense, and apically located. In contrast, fragmentation and shrinkage of nuclei, cytoplasmic vacuoles, mild swelling of rER, large numbers of lysosomes, lipid droplets in the basal cytoplasm, mitochondrial swelling, disorientation, shortening and disorganization of cristae were present in the diabetic groups. The secretory granules were smaller in size and were more homogenous and acinar cells of diabetic rats exhibited variability in the density and structure of secretory granules. DM + ART (50 mg/kg) intervention group had abundant rER, and mitochondrial cristae were visible, while the nucleus had no apparent abnormality. DM + ART (100 mg/kg) had fragmented shrinkage of nuclei, and cytoplasmic vacuoles were observed. Mitochondria were more developed and lysosomes were visible in the cytoplasm. DM + INS intervention groups had nuclear pyknosis, and mitochondria were abundant, while autophagy lysosomes, numerous lipid droplets, and medullary structures were observed in the cytoplasm (Fig. 9).

## Immunohistochemistry: ART decreased protein levels of NFκB1 and TNF-α axis of inflammation induced by DM in the lacrimal gland tissue

**Figure 10:**
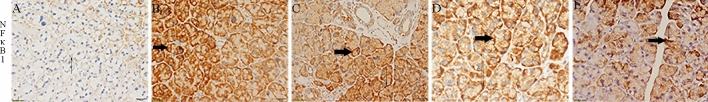
Immunohistochemistry of NFκB1 in the lacrimal gland of rats. (400 ×). In (**A**) healthy control group, (**B**) diabetic group, (**C**) DM + ART (50 mg/kg), (**D**) DM + ART ( 100 mg/kg), (**E**) DM + INS (6 U/kg). NFκB1 was highly expressed in diabetic exorbital acinar cells and was concentrated on the apical membrane and cytoplasm (black arrow). DAB, Scale bar = 20 μm. Original magnification: 400 × (**A**–**E**).

**Figure 11:**
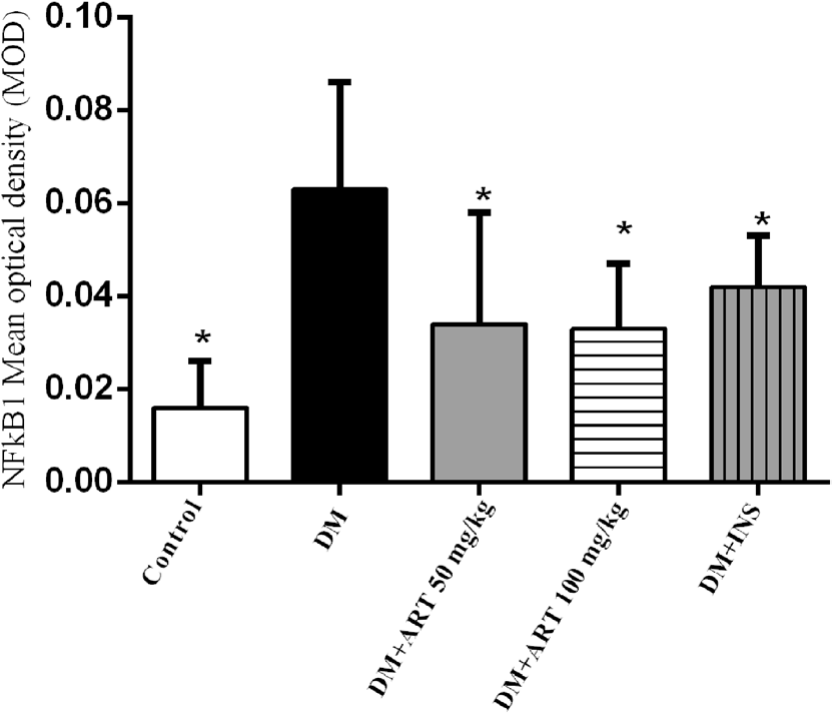
Immunohistochemistry of NFκB1 in the lacrimal gland of rats. DM + ART (100 mg/kg) (**D**) also significantly reduced the expression of NFκB1. Error bars represented SD. A column represents mean ± SD,*P < 0.01 compared with the diabetic group.

**Figure 12:**
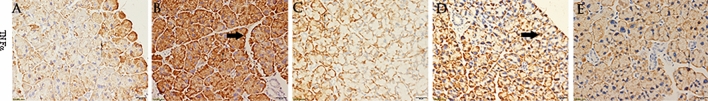
Immunohistochemistry analysis of TNF-α in the lacrimal gland of rats. TNF-α was highly expressed in diabetic exorbital acinar cells and was concentrated on the nucleus and cytoplasm (black arrow). DAB, Scale bar = 20 μm. Original magnification: 400 × (**A**–**E**).

**Figure 13:**
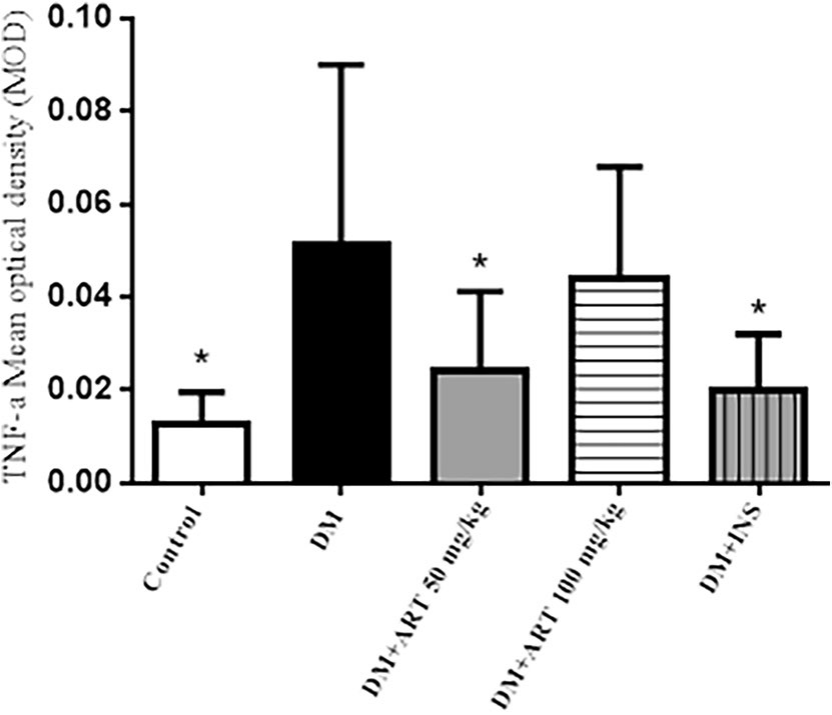
In DM + ART (50 mg/kg) down-regulation of TNF-α in the lacrimal glands of DM rats was significant (^*^P > 0.05). Error bars represented SD. A column represents mean ± SD,*P < 0.01 compared with the diabetic group.

Increased inflammation-related cytokine expression was found in the diabetic lacrimal gland, and NF-κB1 and TNF-α were selected as biomarkers for further immunohistochemistry analysis. The expression levels of NFκB1were localized in the apical membrane and cytoplasm (Figs. 10, 11) and TNF-α was localized in the nucleus and cytoplasm (Figs. 12, 13) in the lacrimal gland tissues of DM rats were significantly higher than those of healthy rats. The expression levels of NFκB1and TNF-α in the lacrimal gland tissues of DM rats were significantly lower than those in ART and INS intervention groups. The expression of NFκB1 was also found significantly decreased in the DM + ART (100 mg/kg) group in the lacrimal glands, but the down-regulation of TNF-α in the DM + ART (50 mg/kg) group and DM + INS group was statistically significant (^*^P > 0.05) (Table 3).

**Table 3:** The immunohistochemistry expression of NFκB1 and TNF-α in the lacrimal gland of rats in each group (x ± s).

Group	$\overline{x}$± s	
	NFκB1 (Average optical density)	TNF-α (Average optical density)
Control	0.016 ± 0.010*	0.012 ± 0.007*
DM	0.063 ± 0.023	0.05 ± 0.039
DM + ART 50 mg/kg	0.034 ± 0.024*	0.024 ± 0.017*
DM + ART 100 mg/kg	0.033 ± 0.014*	0.04 ± 0.024
DM + INS	0.0042 ± 0.011*	0.02 ± 0.01*

## RT-PCR: ART inhibited mRNA levels of NFκB1 and TNF-α axis of inflammation induced by DM in the lacrimal gland tissue

**Figure 14:**
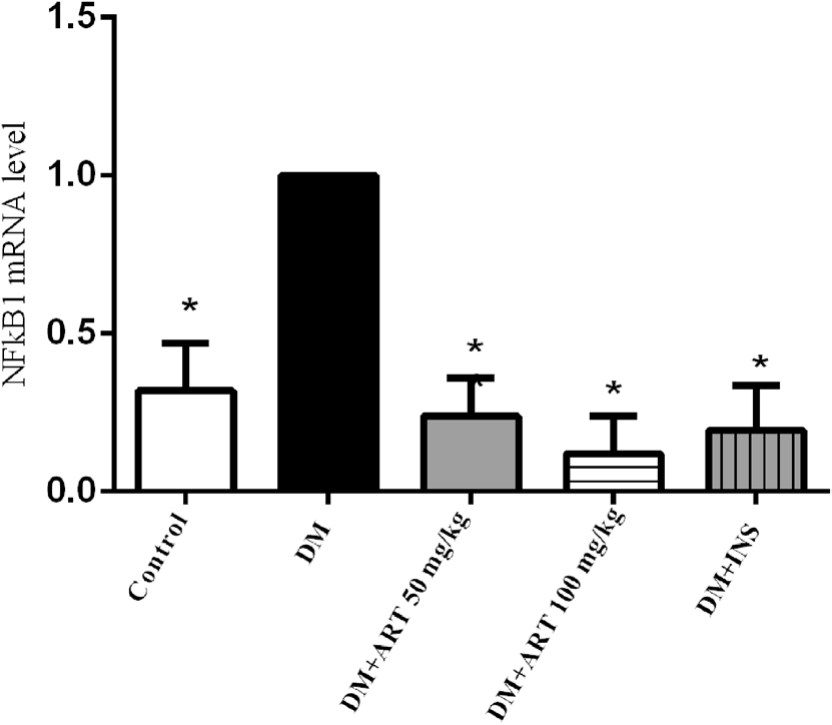
RT-PCR analysis of NFκB1 in the lacrimal gland of rats in each group. Error bars represented SD. A column represents mean ± SD,*P < 0.01 compared with the diabetic group.

**Figure 15:**
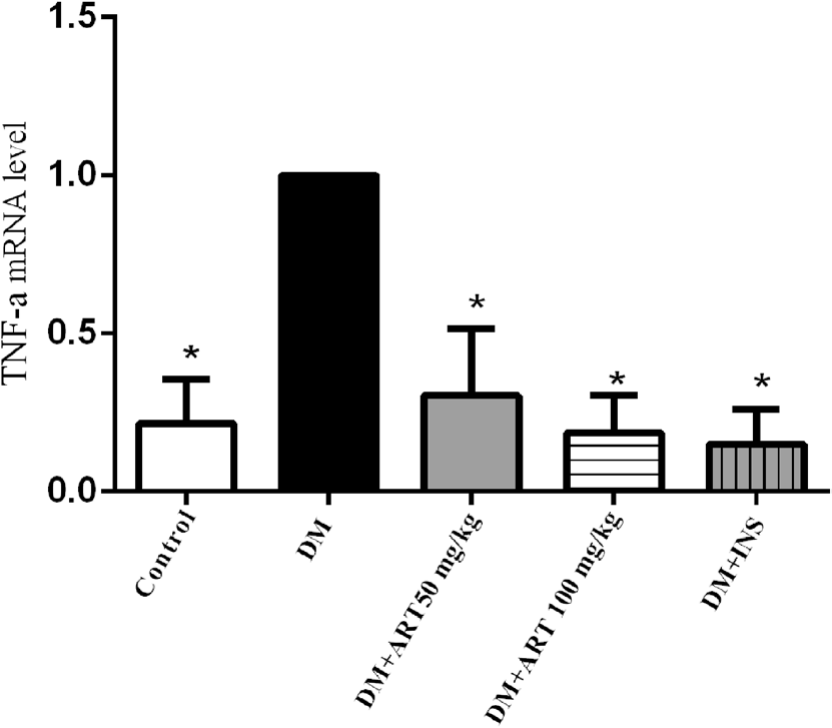
TNF-α mRNA in the lacrimal gland of rats in each group. Error bars represented SD. A column represents mean ± SD,*P < 0.01 compared with the diabetic group.

To further confirm the immunohistochemistry results, we performed real-time PCR analysis. The mRNA levels of inflammatory factors NFκB1 (Fig. 14) and TNF-α (Fig. 15) in lacrimal gland tissues of DM rats were significantly higher than those of healthy rats. The mRNA levels of NFκB1and TNF-α decreased significantly after insulin and ART intervention (^*^P < 0.05) (Table 4).

**Table 4:** Relative expression of NFκB1 and TNF-α in lacrimal gland of rats in each group.

Group	$\overline{x}$± s	
	NFκB1	TNF-α
C	0.32 ± 0.15*	0.23 ± 0.14*
DM	1.0 ± 0	1.0 ± 0
DM + ART 50 mg/kg	0.24 ± 0.12*	0.30 ± 0.21*
DM + ART 100 mg/kg	0.12 ± 0.12*	0.19 ± 0.12*
DM + INS	0.19 ± 0.14*	0.15 ± 0.11*

## Discussion

Diabetes mellitus is a commonly seen metabolic disorder globally and its prevalence continues to rise^17^. It is associated with a range of complications, such as diabetic retinopathy, neuropathy, etc. The treatment options for these complications are limited, prompting researchers to focus on aiming to address this gap. DED syndrome, associated with dysfunction of the corneal layers and lacrimal glands, is a complication of diabetes^18^. STZ is an antibiotic known for destroying pancreatic β-cell, so it is commonly used in scientific research to induce diabetes in animal models^19^. This study proposes that the STZ-induced diabetic rat is a good model for diabetes due to the manifestation of various symptoms in a short period, including hyperglycemia and reduced body weight.

In recent years, there has been a notable rise in the prevalence of diabetic dry eye, making it a significant concern for both mental and physical health^20^. Understanding the molecular mechanisms behind the reduction of tears and the development of dry eye has presented significant challenges. Studies in rats have demonstrated various changes in the lacrimal gland and ocular surface as a result of diabetes^21^. These changes include reduced tear secretion, dysfunction of the salivary gland after 4–5 weeks, corneal epithelial alterations, and histological destruction of the retina after 40 weeks^22^. To further understand, the present study aimed to compare the impact of ART intervention on the lacrimal gland in diabetic rats. The tear secretion in the DM + ART (100 mg/kg) intervention group was increased compared to the DM + ART (50 mg/kg) and DM + INS intervention group in the 4^th^ week of intervention. The data from our study confirmed that DM reduces the weight of LG and directly affects the morphology of LG and other ocular surface tissues such as cornea, lens, and retina.

ART has been found to have a wide range of effects beyond just its antimalarial properties. It has been reported to have secondary effects such as reducing blood sugar levels, as well as exhibiting antitumor, anti-inflammatory, and antifibrotic properties^23,24^. Yu et al.’s research has demonstrated that ART can effectively inhibit the NF-κB signaling pathway and play a regulatory role in the signaling pathway mediated by it^25^. Earlier research has determined that short-term exposure (24 h) to IL-1β defective beta-cell function and long-term exposure (72 h) to IL-1β-induced apoptosis of beta-cells. More importantly, it has validated that ART can protect pancreatic beta-cells in opposition to cytokine-induced impairment of GSIS and apoptosis. These consequences are based on the regulation of SIRT1 in the NF-κB signaling pathway^26^. The primary drug utilized for the clinical management of type 1 diabetes and its associated complications is INS. In this experiment, the effectiveness of ART and INS intervention groups was compared in rats with experimental-induced diabetes models.

Current treatments for dry eye typically focus on relieving symptoms without addressing the underlying cause. While medications like cyclosporine and steroid hormones have been cyclosporine and steroid hormones were developed to inhibit the immune response and treat dry eye, they can lead to adverse effects such as impairment of the eye’s natural defense barrier and secondary infections^27^. There is limited research on traditional Chinese medicine interventions for dry eye. Bidens pilosa L. (*Bp)* extracts have shown anti-inflammatory properties by preventing the production of pro-inflammatory cytokines^28^. The DM group exhibited abnormal lobular patterns and disorganized acinar and ductal cell size and shape as well as infiltration of polymorphonuclear cells and lymphocytes. However, the DM + ART (50 mg/kg) and DM + INS intervention groups displayed normal lobular patterns and no acinar atrophy and fibrosis. The relationship between changes in cornea thickness and diabetic dry eye remains unclear, but studies have demonstrated that reduced corneal epithelial thickness is a diabetic complication^29–31^ We have observed corneal epithelial staining was improved significantly with ART treatment. ART has also been shown to alleviate inflammatory cell infiltration in the lacrimal gland, increased cell nuclei, and fewer vacuoles were seen in the cortical region of the lens.

Mitochondrial dysfunction has been linked to the onset of diabetic complications, such as dry eye disease. The precise reasons for mitochondrial dysfunction in diabetes are still not fully understood. Studies, both in vivo and in vitro, have suggested that the overproduction of reactive oxygen species and oxidative stress may contribute to dry eye disease^32,33^. This is consistent with the observation that changes in mitochondrial structure and function are associated with metabolic disorders, particularly those involving oxidative stress. Research has indicated that severe mitochondrial dysfunction in the lacrimal glands could lead to early diabetic dry eye, while the application of specific mitochondria-targeted antioxidants has promising results in reducing the severity of this condition^34^. These findings advocate for the alleviation of mitochondrial dysfunction as a potential new approach for treating dry eye disease. Our findings revealed that the diabetic group showed striking disturbances in the mitochondrial architecture, together with fragmentation and shrinkage of nuclei, loss of nucleus formation, and condensed chromatin. In contrast, the ART intervention group was well-oriented, and an abundance of mitochondria was present.

Persistent high levels of glucose in the body can lead to chronic oxidative stress, which is a major factor in the development of lacrimal complications in individuals with diabetes^35^. The involvement of salicylates and aspirin, particularly in inhibiting the pro-inflammatory NF-kB signaling pathway, has been identified as a potential mechanism to address this issue^21^. Additionally, the accumulation of glycation products in periodontal tissues due to high glucose levels triggers an inflammatory response, leading to connective tissue damage and bone resorption, exacerbating periodontal inflammation. Additionally, elevated blood sugar levels lead to an inflammatory reaction that triggers both the natural and learned immune systems of the lacrimal functional unit. Moreover, high blood sugar levels result in increased tear film osmolarity, which in turn causes hyperosmolarity in the cells of the ocular surface epithelium and sets off a series of inflammatory reactions that involve MAP kinases and NFkB signaling pathways^36^. This inflammatory response is further supported by the significantly higher expression of NF-kB and TNF- α in diabetic individuals, indicating their crucial role in the inflammatory response in the lacrimal gland induced by diabetes mellitus^37^. Consequently, it is suggested that acinar cells can be protected from TNF- α induced destruction through the NF-kB signaling pathway.

One major limitation of this time-wise changes in the lacrimal gland and tear secretion with control, diabetes, and intervention groups. Therefore, further investigations are warranted. The long-term administration of ART and its efficacy on different degrees of dye eye should be further investigated. Although further studies of the mechanisms are still necessary, our findings suggest that *ART* may be further developed as a therapeutic agent to treat dry eye associated with diabetes mellitus.

## Conclusions

In conclusion, the present study showed morphological alterations in the lacrimal gland and ocular surface tissues including the cornea, lens, and retina in an animal model of diabetic rats. The specific mechanism may be through the activation of the NF-κB signaling pathway and its downstream inflammatory factors. ART intervention can inhibit the NF-κB signaling pathway, and downregulate inflammatory markers, such as TNF-α. However, the regulatory mechanism underlying ART has only been preliminarily explored, and further in-depth mechanistic studies are needed to explore to fully understand its therapeutic effects to improve the quality of life individual diabetes-related ocular issues.

## Materials and methods

### Animal resources and raises

Forty 6-week-old male Sprague–Dawley rats (body weight 200–220 g), were provided by the Animal Experimental Center of Guangxi Medical University, (experimental animal produce license: SCXK GUI 2014–0002, experimental animal application license: SYXK GUI 2014–0003). All animals were kept under constant conditions (at 20 ± 2 °C) and in a 12 h light/dark cycle. Animals were fed with a standard diet and water ad libitum. The study was also carried out in compliance with the ARRIVE guidelines. All animals were raised strictly following the international ethical guidelines and the National Institutes of Health Guide concerning the care and use of laboratory animals. The experiments were carried out with the approval of the Animal Care & Welfare Committee of Guangxi Medical University, (approval No: 201802017).

## Equipment and reagents

Streptozotocin (STZ) and sodium pentobarbital were obtained from Sigma, USA. ART injection was procured from Guilin Pharmaceutical. Rabbit anti-mouse NF-κB and TNF-α polyclonal antibodies were purchased from Abcam Company, UK. Goat anti-rabbit/mouse universal horseradish peroxidase (HRP)-labeled secondary antibody was obtained from Wuhan Doctoral Technology Co., Ltd., China. The pre-stained protein marker, reverse transcription kit, and quantitative polymerase chain reaction (PCR) instrument were purchased from Thermo. Insulin injection was obtained from Xuzhou Wanbang, China. The precision pH test paper URIT 8 V was obtained from Changzhou Oak, China. Phenol Red cotton thread was purchased from Tianjin Jingming, China. RNAiso Plus, SYBR Fluorescence Quantitative PCR kit, and reverse transcription kit were purchased from TaKaRa, Japan.

## Experimental diabetic models

The diabetes model was induced by intraperitoneal injection of Streptozotocin (STZ) at a dosage of 60 mg/kg /body weight dissolved in 0.1 mol/L citric acid buffer (pH 4.4). The healthy control (HC) group (n = 8) was injected with sodium citrate buffer only. The fasting blood glucose levels were detected on the 3rd, 7th, and 14th day with a glucose meter test to verify diabetic status. Fasting hyperglycemia over more than 16.7 mmol/L indicates a diabetic model. On the 15^th^ day, diabetic groups were further divided into four groups (n = 8): Diabetic (DM) group, diabetic with 50 mg/kg gavage of ART (DM + ART 50 mg/kg), diabetic with 100 mg/kg gavage of ART (DM + ART 100 mg/kg), and diabetic with insulin intervention (6 U/kg body weight, a daily subcutaneous injection) group (DM + INS). Throughout the study, for four consecutive weeks, the fasting blood glucose and body weight were detected every week from each experimental-induced diabetic rat indicating hyperglycemia. A tear secretion test was done, and tissues were harvested after animals were sacrificed at the end of 4^th^ week of intervention. After the treatment, the rats were euthanized via excess anesthesia with pentobarbital sodium overdose (intraperitoneal injection; Sigma, USA).

## Measurement of aqueous tear production

Tear secretion was measured using phenol red-impregnated cotton threads at the end of the experiment which is on the 4th week of intervention. Tear volume was measured once at the end of the experiment. The threads were held with jeweler forceps and placed between the lower lid and the globe for 30 s. The wetting of the thread was measured with a ruler with 0.5 mm precision secretion^17^.

## H&E staining

Eyeball and exorbital lacrimal glands were harvested immediately after animals were sacrificed and fixed in 10% neutral buffered formalin solution for 24 h and then processed paraffin-embedded sliced (5 µm thick). The slides were dewaxed in xylene for 4 min per cylinder, dehydrated by gradient alcohol for 5 min, slightly washed with distilled water 4 times, 1 min each time, stained with hematoxylin dye for 5 min, slowly washed in flowing water for 3 s, acidified with 1% hydrochloric acid for 2 s, slowly washed in flowing water for 15 s, slowly buffered in distilled water for 3 s, and stained with 0.5% eosin for 4 min and after dried finally mounted under coverslips and then observed under a light microscope.

## Immunohistochemistry analysis

The slides were dewaxed in multiple dewaxing cycles in xylene, for 4 min per cycle, dehydrated by gradient alcohol for 5 min, and slightly washed with distilled water four times, 1 min each time. Heating was applied for 20 min to induce antigen retrieval in 10 mM sodium citrate buffer pH 6.0. Endogenous peroxide activity was quenched using 3% hydrogen peroxide in absolute methanol for 10 min at room temperature (RT). The tissue sections were rinsed thrice with PBS (pH 7.4) for 5 min between each consecutive step. The parts were then incubated in goat serum (blocking serum) for 5 min to prevent nonspecific antibody binding.

After that, the sections were then incubated with a primary antibody, rabbit anti-mouse NFκB1 polyclonal antibody (concentration of 1:1000), or rabbit anti-mouse TNF-α (concentration of 1:2000) antibody, overnight at 4 degrees Celsius in a humidified chamber. After being washed with PBS, the sections were treated with a peroxidase-conjugated rabbit secondary antibody for 45 min at room temperature and then washed with PBS again. The reaction products were developed with a mix of DAB Tris tablets and 0.3% H_2_O_2_. Nuclear staining was performed by treating with hematoxylin for 2 s. The sections were then mounted and examined. A double-blind method at high magnification (400 ×), was used to get photographs of tissues and store them in the computer, and Image Pro PlusPlus 6.0 Version professional image analysis software (https://mediacy.com/image-pro/) was used to analyze images^13^.

## Transmission electron microscopy (TEM)

Exorbital LG tissues from the five groups that were fixed for TEM were rinsed in 3% glutaraldehyde. Three samples were randomly selected from each group. Within 60 s after the execution, a sharp scalpel was used to rapidly cut the lacrimal gland tissue block of about 1 mm^3^ on the ice and rapidly put it into the fixing solution for low-temperature shading and preservation. Specimens were fixed 24 h in 2.5% glutaraldehyde, 2 h in 2% osmic acid, gradient dehydrated in 50%, 70%, 90%, 100% acetone within 2 h, dipped in 37 ℃ epoxy resin soak solution (epoxy resin: acetone = 1:1) for 24 h, embedded in epoxy resin. Semi thin sections and ultrathin sections were processed and electronic double dyeing by uranyl acetate and lead nitrate on grides, and observed under a transmission electron microscope.

## Quantitative real-time PCR analysis

Total RNA was extracted from lacrimal glands with TRIzol. First-strand complementary DNA (cDNA) was synthesized from 2 µg of total RNA using oligo (dT) primers and M-MLV reverse transcriptase. The PCR reaction was performed in a 20 µl volume with PCR downstream primer (10 μM) 0.8 μL; cDNA 1.0 μL; RNase-Free dH2O 7.0 μL. SYBR-Green (1:20,000 dilution) was included in each reaction to allow relative quantification of RNA levels using the ABI StepOne Plus detection system. Real-time PCR was then performed for primers designed to amplify NFκB1 (Forward: 5'- GCT GCC AAA GAA GGA CAC GAC A-3'; Reverse: 5'-GGC AGG CTA TTG CTC ATC ACA G -3') TNF-α (Forward: 5'- CCC GCA TCC CAG GAC CTC TCT-3'; Reverse: 5'-CGG GGG ACT GGC GA-3') using GAPDH (Forward: 5'-GACATGCCGCCTGGAGAAAC-3'; Reverse: 5 ‘-AGCCCAGGATGCCCTTTAGT -3') as an internal control. The reaction was cycled through the following conditions: on holding stage for 30 s at 95 °C, on cycling stage denaturation for 5 s at 94 °C, annealing for 34 s at 60 °C, the extension for 15 s at 95 °C, and a final extension at 60 °C for 10 min, 40 cycles. The recorded data was then analyzed using the ΔΔCt method. The change ratio for a specific gene was obtained by calculating ΔCt = Ct [target gene] − Ct [internal control], ΔCt [samples] ΔCt [Control] = ΔΔCt. Finally, relative quantification (RQ) was calculated using the formula 2^-ΔΔCt^^36^.

## Statistical analysis

Data were analyzed using SPSS version 22.0. All data are expressed as mean ± standard deviation (SD). Statistical comparisons between the groups were done using one-way analysis of variance (ANOVA) followed by Tukey’s test. Differences were considered statistically significant at *P* < 0.05^36^.

### Ethics approval and consent to participate

All experiments were performed according to the Chinese legislation on the protection of animals and the National Institutes of Health (NIH), Guide for the Care and Use of Laboratory Animals (Institute of Laboratory Animal Resources, Animal Guide from Guangxi Medical University, Animal experiment center), and ethics approval of Animal Care & Welfare Committee of Guangxi Medical University (No. 201802017) . The local governmental animal protection committee approved them.

## Data Availability

The datasets generated and/or analyzed during the current study are available from the correspondence author and details are mentioned above.

## Abbreviations

DM: Diabetes mellitus
DES: Dry eye syndrome
TDDE: Tear deficient dry eye disease
DED: Dry eye disease
OS: Ocular surface
LG: Lacrimal gland
ART: Artesunate
GSIS: Glucose stimulates insulin secretion
GABA: Activation of the g-aminobutyric acid
ROS: Reactive oxygen species
RQ: Relative quantification
SD: Standard deviation
BUT: Tear film break up time
